# Editorial: The role of red blood cells in the immune response of fish

**DOI:** 10.3389/fimmu.2022.1005546

**Published:** 2022-09-06

**Authors:** Maria del Mar Ortega-Villaizan, Julio Coll, Espen Rimstad

**Affiliations:** ^1^ Departamento de Bioquímica y Biología Molecular, Instituto de Biología Molecular y Celular (IBMC), Universidad Miguel Hernández (UMH), Elche, Spain; ^2^ Departamento de Bioquímica y Biología Molecular, Instituto de Investigación, Desarrollo e Innovación en Biotecnologîa Sanitaria de Elche (IDiBE), Universidad Miguel Hernández (UMH), Elche, Spain; ^3^ Departamento de Biotecnología, Instituto Nacional de Investigación y Tecnología Agraria y Alimentaria (INIA), Madrid, Spain; ^4^ Faculty of Veterinary Medicine, Norwegian University of Life Sciences, Oslo, Norway

**Keywords:** red blood cells, erythrocytes, fish, immune response, virus, prophylactics, transcriptomic, proteomic

The role of nucleated red blood cells (RBCs) as cellular mediators of the immune response in fish has emerged as a hot research subject that has attracted fish immunologists’ attention during the past decade. Fish are the most primitive vertebrates, possessing many of the cell types and molecules found in higher vertebrates. However, the immune system of fish is peculiarly dissimilar to that of higher vertebrates. Immune response is still poorly understood in most fish species, particularly in species recently introduced to aquaculture. These facts have limited the development of strategies to combat infectious diseases so far. Concerning fish RBCs, a number of biological processes relevant to immunity had been described for them: (i) pathogen recognition; (ii) clearance of pathogens by means of binding microbial immune complexes and (iii) production of cytokines or specific signalling molecules in response to pathogens. This Special Issue aimed to gather new research findings and advances on the role of RBCs in mediating the immune response in fish, with the hope that this forum could foster further collaborations in this emerging area of research.

In this Research Topic, the advances on the immune response of fish RBCs to viruses or to different antiviral prophylactics will be presented. It contains articles related to the RBCs immune response to RNA and DNA viruses, as well as to different types of prophylactics, such an antigen-based DNA vaccine or a nanosctructured recombinant antigenic protein.

## Fish red blood cells role played in the immune response to virus

Salmonid red blood cells are the primary target cells for Piscine orthoreovirus (PRV), a double-stranded RNA (dsRNA) virus that belongs to the Reoviridae family. Wessel et al. reviewed how the three genotypes of PRV (PRV-1,2,3) infect several salmonid species (Atlantic salmon, Chinook salmon, Coho salmon, rainbow trout and brown trout), and cause diseases like heart and skeletal muscle inflammation (HSMI) or erythrocyte inclusion body syndrome (EIBS). During the early stage of infection, salmonid erythrocytes, or red blood cells (RBCs), are the primary virus-replicating cells further contributing to virus dissemination within the host. In this initial stage, cytoplasmic “virus factories” could be observed in RBCs, and these were shown to be the primary sites for the formation of new virions. The authors showed how Atlantic salmon PRV-infected RBCs mounted a strong long-lasting innate antiviral response for several weeks after the beginning of infection. The antiviral response correlated with increasing PRV levels in RBCs demonstrating that cell response and viral replication were linked in the early phase. This RBCs antiviral response implied the upregulation of genes associated with immune suppression, IFN-related genes and potential inhibitors of translation, halting PRV-1 protein production in RBCs.

Viral hemorrhagic septicemia virus (VHSV) is a single-stranded RNA (ssRNA) which belongs to the Rhabdoviridae family. Nombela et al. (1) previously described that VHSV infection appears to be halted in rainbow trout RBCs, pointing to diverse mechanisms responsible for the antiviral immune response of rainbow trout RBCs to VHSV. Chico et al., in an attempt to identify the specific rainbow trout RBC proteins that interact directly with VHSV, characterized the immunoprecipitated (IP) proteome of RBCs exposed to VHSV. The IP proteomic characterization identified 31 proteins by mass spectrometry analysis. Among them, the authors identified the interferon-induced protein with tetratricopeptide repeats 5 (IFIT5), a protein that belongs to a family of proteins that are induced after the production of type I interferon. The authors confirmed the contribution of IFIT5 in the rainbow trout RBC antiviral response. They detected a relationship between the peak of IFIT5 expression level and the decay in VHSV replication. In addition, silencing the *ifit5* gene by means of siRNA resulted in a significant increase in VHSV replication in RBCs. Moreover, a proximity ligation assay indicated a protein colocalization of IFIT5 with the glycoprotein G of VHSV. Taken together these findings suggested a role for IFIT5 in the antiviral response of RBCs against VHSV.

Rock bream iridovirus (RBIV), a double-stranded DNA (dsDNA) virus that belongs to the Iridoviridae family, causes severe mass mortality in Korean rock bream (*Oplegnathus fasciatus*) populations. Jung et al., in an effort to unveil the immune defense mechanisms of rock bream against RBIV, explored the involvement of rock bream RBCs in the immune response against RBIV. They found that RBIV copy number in RBCs gradually increased from 4 days post-infection (dpi), peaking at 10 dpi. They identified 318 proteins significantly regulated in RBCs from RBIV-infected fish, 183 proteins upregulated and 135 downregulated. Differentially upregulated proteins were mainly involved in the MHC class I-related protein pathway, cellular detoxification, cellular amino acid metabolic processes, spliceosome, and snRNP assembly. They also found the regulation of apoptosis-related proteins, such as caspase-6 (CASP6), caspase-9 (CASP9), and Fas cell surface death receptor (FAS). Interestingly, the expression of genes related to the ISG15 antiviral mechanism pathway, were found downregulated in RBCs from RBIV-infected individuals. Overall, their findings contributed to a better understanding of RBIV pathogenesis and virus-host interaction.

## Fish red blood cells role played in the immune response elicited by antiviral vaccines

Nowadays, vaccination is the primary way to control and prevent viral diseases in aquaculture. The advancement on novel vaccination methods is a pivotal point in fish vaccinology. Nanostructured recombinant proteins have recently emerged as a promising new strategy. Nanostructured cytokines were previously shown to immunostimulate and protect fish against bacterial infections (Thwaite et al.). Puente-Marin et al. explored the role played by RBCs in the immune response to two nanostructured recombinant proteins, TNFα and a fragment of the glycoprotein G of VHSV (G-VHSV). Different *in vitro* and *in vivo* assays were performed to demonstrate, for the first time, that rainbow trout RBCs were able to endocytose the nanostructured TNFα and G-VHSV protein fragment *in vitro*, in spite of RBCs not being typical phagocytic cells. Also, in response to nanostructured TNFα and G-VHSV fragment, the authors showed the modulation of the expression of different immune genes related to cytokine signalling, antigen presentation, IFN-signalling and oxidative stress, demonstrating the role of rainbow trout RBCs in the immune response triggered by antigenic proteins.

DNA immunization is known as one of the best strategies to control and prevent viral diseases in aquaculture. DNA vaccines based on the rhabdoviral glycoprotein G gene have been shown to be effective against fish rhabdoviruses. Nevertheless, better knowledge about the immune response triggered by DNA vaccines is necessary to advance on more effective strategies. Puente-Marin et al. investigated the role of fish RBCs in the immune response triggered by a DNA vaccine. They showed, for the first time, that rainbow trout RBCs could express the G-VHSV after *ex vivo* transfection with the DNA vaccine. They found that RBCs could modulate the expression of immune genes and proteins in response to the G-VHSV DNA vaccine. Transcriptome profiling of RBCs expressing the G-VHSV revealed changes in gene expression related to G-protein coupled receptor (GPCR)-downstream signalling or complement activation. The proteomic analyses revealed proteins involved in interferon-stimulated gene 15 (ISG15) antiviral mechanisms, antigen presentation of exogenous peptides, and the proteasome. Moreover, they showed how conditioned medium from G-VHSV-transfected RBCs conferred antiviral protection in RTG-2 cells infected with VHSV. Overall, the authors demonstrated that rainbow trout RBCs could be actively taking part in the fish immune response to a GVHSV DNA vaccine.

In summary, the scientific advances covered in this Research Topic greatly contribute to shed light into the mechanisms that are triggered in fish RBCs in response to the viral infection and to vaccination.

## Author contributions

MO-V wrote the manuscript. JC, ER and MO-V contributed to conception of the Research Topic. All authors contributed to the article and approved the submitted version. This work was supported by the European Research Council fund to MO-V (ERC Starting Grant GA639249)

## Funding

This work was supported by the European Research Council fund to MO-V (ERC Starting Grant GA639249).

## Conflict of interest

The authors declare that the research was conducted in the absence of any commercial or financial relationships that could be construed as a potential conflict of interest.

## Publisher’s note

All claims expressed in this article are solely those of the authors and do not necessarily represent those of their affiliated organizations, or those of the publisher, the editors and the reviewers. Any product that may be evaluated in this article, or claim that may be made by its manufacturer, is not guaranteed or endorsed by the publisher.

## References

[1] Nombela I Puente-Marin S Chico V Villena AJ Carracedo B Ciordia S. Identification of diverse defense mechanisms in rainbow trout red blood cells in response to halted replication of VHS virus. F1000Res (2017) 6:1958. doi: 10.12688/f1000research.12985.1 29527292 PMC5820608

